# Supplementary material for: Long-term microgravity experiments reveal a new mechanism for particle aggregation in suspension
Source: NPJ Microgravity. 2025 Sep 9;11:63. doi: 10.1038/s41526-025-00523-7 (PMC12420831; doi:10.1038/s41526-025-00523-7)
Supplement: Supplementary file 1 — Supplementary Information [file 41526_2025_523_MOESM1_ESM.pdf]

Supplementary information

Supplementary Note 1: Microgravity Experiments

The experiment began on July 26, 2018. Due to technical problems with the flash unit, the run had to be aborted after 13 days and restarted on August 13, 2018. The second run continued for a total of 99 days and was completed on November 20, 2018. The samples were returned on January 14, 2019 as part of the SpaceX CRS-16 mission.

The cuvettes of the microgravity experiments were prepared in an Earth-bound environment for security reasons. Each cuvette was filled with a varying proportion of kaolinite, montmorillonite, and sand, as well as salt water generated by mixing water and sodium chloride. Detailed information on the applied materials can be found in Supplementary Table 1.

Supplementary Table 1: Material properties.

Sample name	Sample P/N
Sodium chloride	746398-2.5KG
Kaolin	18616-5Kg
Montmorillonite	69866-100G
Sea sand 100-315 microns	1.07711.1000/1kg

Each cuvette was sealed on Earth before being transported to the International Space Station (ISS) on a Falcon 9 rocket on the SpaceX CRS-15 mission on June 26, 2018. Upon arrival, the cuvettes were installed in the Binary Colloidal Alloy Test (BCAT) apparatus; a rack, in which ten cuvettes are arranged in a two-by-five array. An image of the setup on board the ISS is given in Supplementary Figure 1. Supplementary Figure 1a shows the entire BCAT system together with a Nikon D2Xs camera that is equipped with a Nikon AF Micro-Nikkor 105/2.8 D lens and a SB-800 flash on the rear side. Supplementary Figure 1b presents a photo of the rack holding the 10 cuvettes, where the numbers on the cuvettes correspond to the listed compositions presented in Table 1 of the Main Article (MA).

Continuous recordings of the g-jitter were taken by the accelerometers SAMS 121f02 and 121f05 that were installed in the vicinity of the experiments in the Japanese Pressurized Module (JPM) of the Japenese Experiment Module (JEM). Supplementary Figure 2 shows the spectra plots of the minimum, maximum, and average accelerations for the associated frequencies. The figures represent from top to bottom the data of the x-, y-, and z-axes. Here, the x-axis is parallel to the center line of the laboratory module, the y-axis is parallel to the starboard truss axis, and the z-axis is orthogonal pointing to the Nadir direction [1]. The figures complement Fig. 1 of MA, where the representations of the x-axis of both sensors are shown again for the sake of completeness.

In addition to the representation of cuvette no. 2 in Fig. 2 of MA, Supplementary Figure 3 illustrates cuvette 7 over the duration of the experiments. The presence of air bubbles in the center of the cuvette at the beginning of the experiments prevented a reliable analysis of the aggregates. As the experiment progressed, the air bubbles migrated towards the magnet, freeing up the center to allow the determination of the aggregate size. Hence, the presentation of the aggregate size over time starts on day 4 for cuvette no. 7 in Fig. 3 of MA. Similar to cuvette no. 2, patterns develop over time that indicate aggregates. The

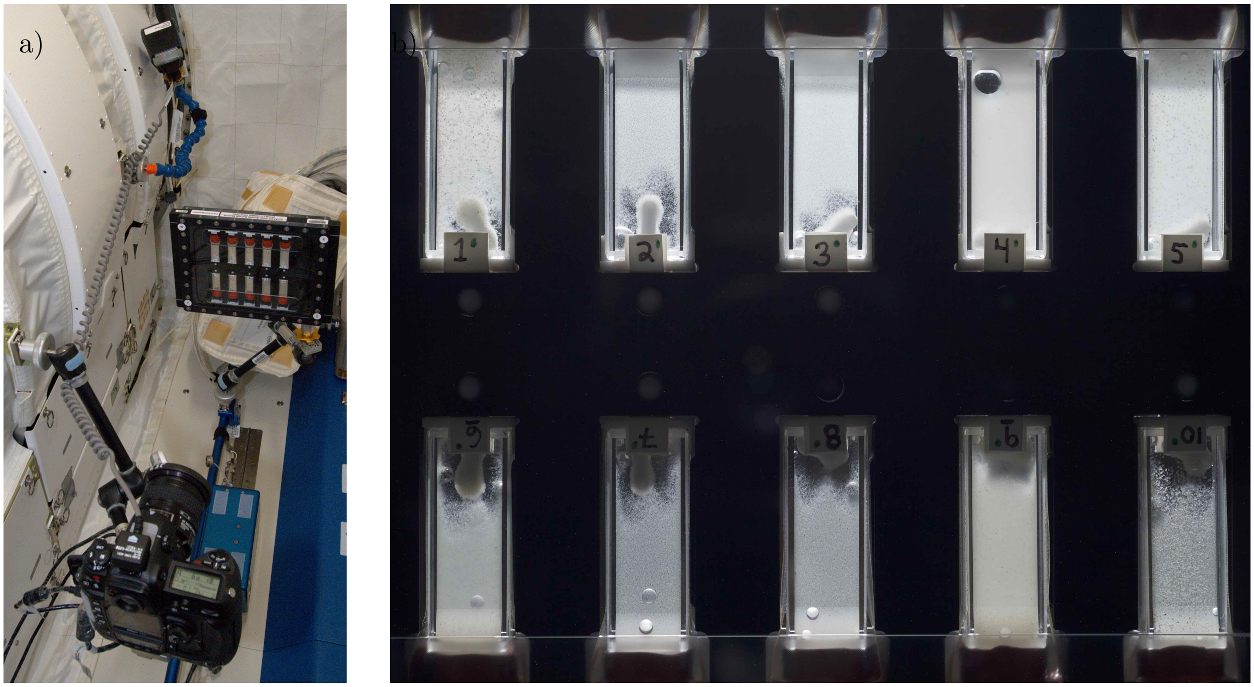

Supplementary Figure 1: Experimental setup with a) the entire BCAT system with image recording equipment and b) a closer view of the cuvettes with the numbering according to Table 1 of MA.

aggregate structures become even more evident in the high-resolution close-ups of the cuvettes presented in Supplementary Figure 4.

Supplementary Note 2: Image Analysis

The image analysis is performed by applying an autocorrelation function in which the 2D intensity autocorrelation C_{2D} is calculated [2, 3]. The analyzed image is considered as an intensity distribution $I(x, y)$ of the gray scale, where x and y are the pixel coordinates. A copy of the same image is taken and shifted by an offset (x_0, y_0) that yields $I(x-x_0, y-y_0)$. The parts overlapping the original and copied shifted images are compared by multiplying their intensity values at each pixel. The result is adjusted by normalizing the product with the squared intensity distribution $I^2(x, y)$ to obtain the similarity of the patterns.

$$C_{2D}(x_0, y_0) = \frac{\sum_{x,y} I(x, y) I(x - x_0, y - y_0)}{\sum_{x,y} I^2(x, y)} \tag{1}$$

The results of C_{2D} are in the range of $[-1, 1]$, which describes a negative and positive correlation, respectively, while $C_{2D} = 0$ represents an uncorrelated signal. We obtain $C_{2D}(0, 0) = 1$ for $x_0 = y_0 = 0$. Based on this, the azimuthal average is calculated, where the results of C_{2D} are averaged around the central point $C_{2D}(0, 0)$ in all radial directions. This results in a one-dimensional radial autocorrelation function C_{1D}.

$$C_{1D}(r) \equiv \langle C_{2D}(x_0, y_0) \rangle_\theta \ . \tag{2}$$

Here, $r \equiv \sqrt{x_0^2 + y_0^2}$ represents the magnitude of the offset and $\langle ... \rangle_\theta$ the azimuthal average in all radial directions $\theta \equiv \arctan(y_0/x_0)$. C_{1D} is used to determine a characteristic length scale that describes the size of an aggregate. The graph of C_{1D} is presented in Supplementary Figure 5. Note that this representation is the same for the autocorrelation function C given in equation (5) of MA. Starting at unity at $r = 0$,

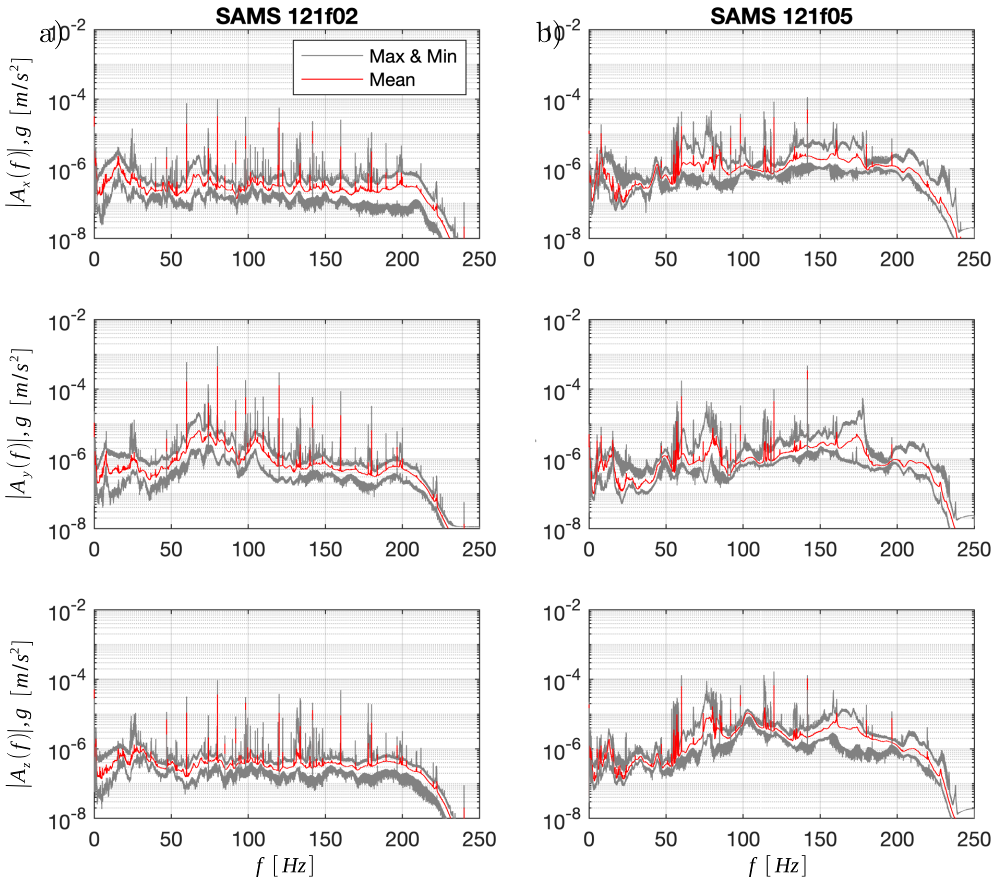

Supplementary Figure 2: Spectra plots of the on board acceleration and frequency ranges of the g-jitter. The acceleration is expressed as a fraction of the gravitational acceleration g. a) presents the data of accelerometer SAMS 121f02 and b) of SAMS 121f05. The upper figures represent the data for the x-, the middle for the y-, and the bottom for the z-axes. The representations of the x-axis are the same as in Fig. 1 of MA and are shown here for the sake of completeness.

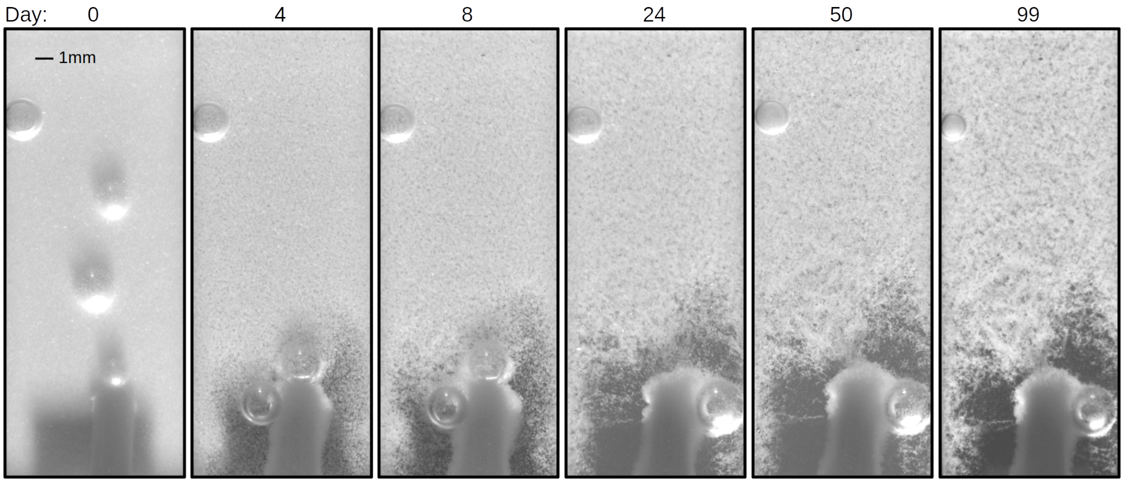

Supplementary Figure 3: Selected photographs of cuvette no. 7 over the duration of the experiment.

C_{1D} decreases to a local minimum at $r = 1.16$ (marked by the dashed vertical line in Supplementary Figure 5) and subsequently increases to reach a local maximum at $r = 1.96$. As indicated by the dashed

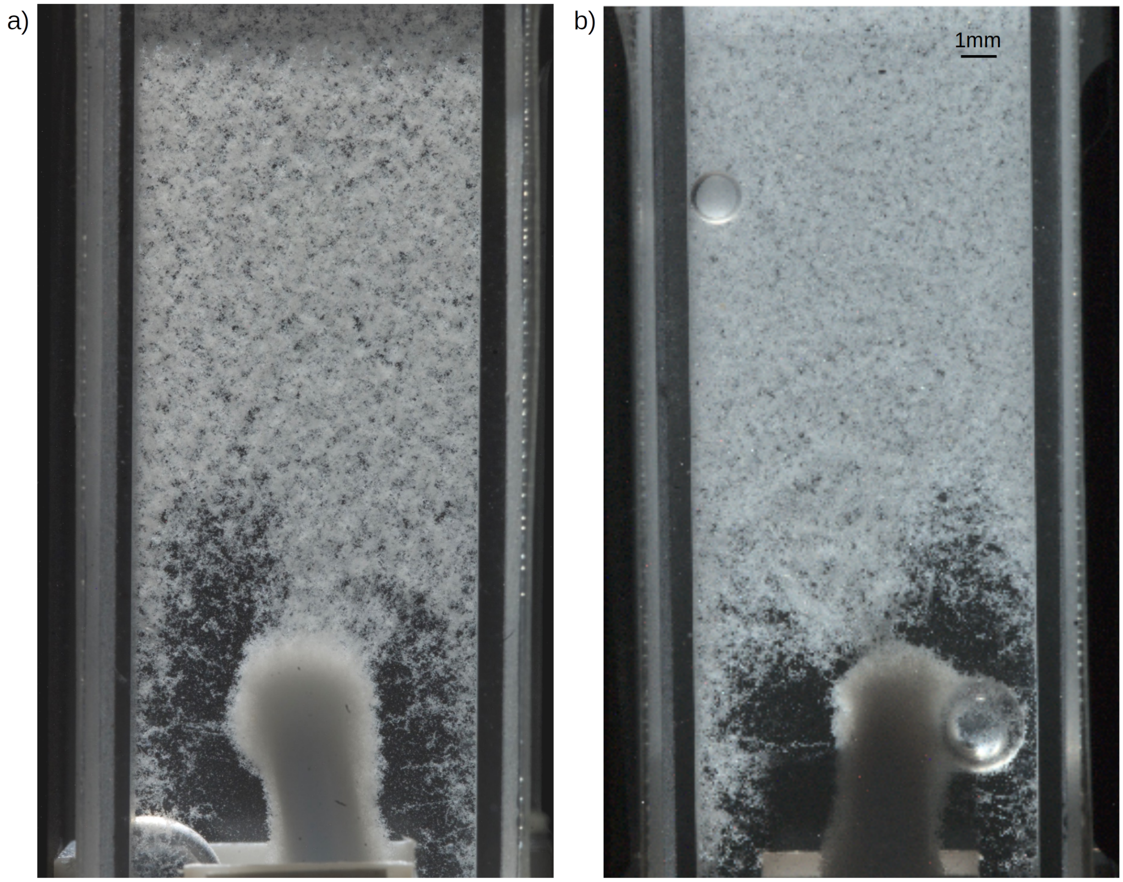

Supplementary Figure 4: High-resolution close-ups of cuvette no. 2 in a) and cuvette no. 7 in b).

line, the radius of the aggregate R_a is defined by the first local trough. The change in this quantity over the course of the experiment thus provides an indication of the overall aggregate growth over time. The aggregate size d_a used in MA is computed by $d_a = 2R_a$.

Supplementary Note 3: Direct Numerical Simulations

Particle-resolved direct numerical simulations (pr-DNS) were performed to investigate the behavior of monodisperse spherical particles with cohesive properties that are submerged in a cubic container of size $L_{x,y,z} = 20d_p$, where d_p represents the particle diameter. The container is filled with fluid and subjected to monochromatic oscillations. We apply a non-inertial frame of reference accounting for the acceleration of the applied external oscillation, as explained in detail in [4]. We apply triple-periodic boundary conditions in all directions and a no-slip condition on the particle surfaces. Fluid-particle interactions are taken into account by applying the immersed boundary method (IBM) [5–7], where the fluid is discretized by a uniform rectangular grid with grid cell size $\Delta x = \Delta y = \Delta z = h$, with a spatial resolution of $d_p/h = 20$, as comprehensively described in [7]. The Navier-Stokes equations and the continuity equation are solved for an incompressible Newtonian fluid:

$$\frac{\partial \mathbf{u}}{\partial t} + \nabla \cdot (\mathbf{u}\mathbf{u}) = -\frac{1}{\rho_f}\,\nabla p + \nu_f \nabla^2 \mathbf{u} + \mathbf{f}_{\mathrm{IBM}}\;, \tag{3}$$

$$\nabla \cdot \mathbf{u} = 0\;. \tag{4}$$

Here, $\mathbf{u} = (u, v, w)^T$ represents the fluid velocity vector in Cartesian components, t the time, ρ_f the

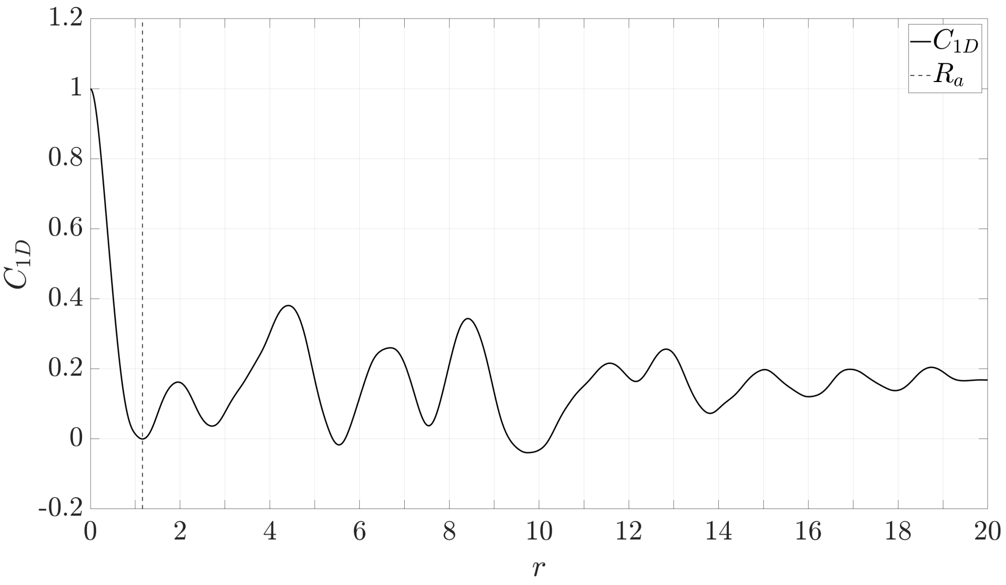

Supplementary Figure 5: The progression of C_{1D} of an exemplary image analysis. The first local trough after $C_{1D}(0) = 1$ indicates the radius of the aggregate and is highlighted by the dashed line.

fluid density, p the fluid pressure, ν_f the fluid kinematic viscosity, and $\mathbf{f}_{\text{IBM}}$ the volume force of the IBM acting on the particle surface to impose the no-slip condition. The $\mathbf{f}_{\text{IBM}}$-term also connects the motion of the particle to the fluid phase. Equations (3) and (4) are integrated by a third-order low-storage Runge-Kutta scheme in time together with a second-order finite difference method in space, respectively. The fast Fourier transform is applied to calculate the pressure correction for continuity using a direct solver.

In the context of the IBM, the motion of each individual spherical particle is calculated by solving the Newton-Euler equations:

$$m_p \frac{\mathrm{d}\mathbf{u}_p'}{\mathrm{d}t} = \mathbf{F}_\mathrm{h} + \mathbf{F}_\mathrm{c} - (\rho_p - \rho_f)\, V_p \frac{\mathrm{d}\mathbf{u}_f}{\mathrm{d}t} \ , \tag{5}$$

$$I_p \frac{\mathrm{d}\boldsymbol{\omega}_p}{\mathrm{d}t} = \mathbf{M}_h \ + \ \mathbf{M}_c \ . \tag{6}$$

Here, m_p, V_p and ρ_p are the mass, volume, and density of the particles, respectively, $I_p = \pi \rho_p D_p^5/60$ the moment of inertia where $\boldsymbol{\omega}_p = (\omega_{p,x}, \omega_{p,y}, \omega_{p,z})^T$ is the angular velocity vector. $\mathbf{u}_p'$ represents the particle velocity in a non-inertial reference frame as derived in [4]. The first terms on the right hand side of (5) and (6), $\mathbf{F}_\mathrm{h}$ and $\mathbf{M}_\mathrm{h}$, denote the hydrodynamic forces and torques, respectively. The fluid-particle interactions can be computed via

$$\mathbf{F}_\mathrm{h} = \oint_{S_p} \boldsymbol{\tau} \cdot \mathbf{n}\, dS \ , \qquad \mathbf{M}_\mathrm{h} = \oint_{S_p} \mathbf{r} \times (\boldsymbol{\tau} \cdot \mathbf{n})\, dS \ , \tag{7}$$

where $\boldsymbol{\tau} = -p\mathbf{I} + \mu_f \left[\nabla \mathbf{u} + (\nabla \mathbf{u})^T\right]$ represents the hydrodynamic stress tensor that includes the identity matrix $\mathbf{I}$ and the dynamic viscosity μ_f of the fluid. Furthermore, $\mathbf{n}$ is the normal vector pointing outward from the particle surface S_p, and $\mathbf{r}$ is the position vector pointing from the particle center of mass to a point on S_p. The forces and torques due to collision are represented by $\mathbf{F}_\mathrm{c}$ and $\mathbf{M}_\mathrm{c}$, respectively. Here, we account for normal and tangential contact, as well as unresolved lubrication forces that emerge when fluid is squeezed out of particle gaps $\zeta \leq 2h$, where ζ the distance between the particle surfaces. Full details on the computation of short-range hydrodynamic as well as collision forces and torques can be found in [7] and [8]. The last term on the right-hand side of (5) represents the transformation of the translational particle motion to the non-inertial reference frame, with V_p the volume of a spherical particle and $\mathbf{u}_f$ the

velocity of the oscillatory fluid as discussed in detail in the Methods of MA. We refer to [4] for further details on the transformation of the reference frame.

We use the cohesive force model of [8] to account for attractive particle-particle interaction. This model is characterized by the non-dimensional cohesive number Co defined as

$$Co = \frac{max(||F_{coh}||)}{m_f\ d_p\ \Omega^2} = \frac{max(||F_{coh}||)}{\rho_f\ \pi\ d_p^3/6\ d_p\ \Omega^2}\ , \tag{8}$$

with the cohesive force

$$F_{coh} = -\frac{A_H\ R_{eff}}{\zeta_0\ \lambda^3}\left(\zeta_n^2 - \zeta_n\lambda\right)\ , \tag{9}$$

with its maximum at $\zeta = \lambda/2$. Inserting this maximum into (9) yields

$$max(||F_{coh}||) = \frac{A_H\ R_{eff}}{\zeta_0\ 4\lambda}\ . \tag{10}$$

Here, m_f represents the fluid mass, $\Omega = 2\pi f$ the angular frequency with f the oscillation frequency, R_{eff} the effective radius defined as $R_{eff} = \frac{R_p R_q}{R_p + R_q}$, with R_p and R_q the radii of the interacting particles p and q, and $\lambda = d_p/20$ the range over which the cohesive force acts. The values for the Hamaker constant $A_H = 10^{-20}\,[J]$ [9] and the microscopic size of the surface asperities of a particle $\zeta_0 = 2 \cdot 10^{-10}\,[m]$ [10] were directly selected from the literature. Applying the values given above together with the parameters stated in MA, we obtain $Co = 0.19$.

The initial placement of the primary particles was random while ensuring that they were not in contact. In this regard, a minimum initial distance $\zeta > 2h$ was applied to avoid lubrication effects from the very beginning of the simulation. Both the fluid and the particles were initially at rest, with the latter free to move in response to the fluid-particle and particle-particle interactions. Each simulation was run for $25,000$ oscillation periods T_o with a time step of $\Delta t = T_o/200$ resulting in a total of $5,000,000$ time steps. The position and velocity data of the particles were stored at every 10th time step. This results in a high-resolution dataset of the particle motion, as it was recorded at 20 points in time within each T_o.

Supplementary Note 4: Derivation of empirical fit

The empirical fit provided by equation (2) of MA represents the growth of a spherical aggregate over time and is derived based on the governing differential equation:

$$\frac{d\ell}{dt} = \frac{D}{\pi\ell} \tag{11}$$

This equation describes the rate of change in the size of the characteristic length of the aggregate considered ℓ over time t depending on the diffusion coefficient D, which is assumed to be constant, and the spherical circumference $\pi\ell$. We separate the variables to solve the differential equation:

$$\pi\ell\, d\ell = D\, dt \quad , \tag{12}$$

which describes that the infinitesimal growth of the circumference is directly proportional to the diffusion flux, which is responsible for the aggregate growth over time. We determine the change in aggregate size as a function of time by integrating the left-hand side with respect to ℓ and the right-hand side with respect to t.

$$\int_{\ell_0}^{\ell} \pi\ell\, d\ell = \int_{t_0}^{t} D\, dt \tag{13}$$

$$\left[\frac{1}{2}\pi\,\ell^2\right]_{\ell_0}^{\ell} = \Big[D\,t\Big]_{t_0}^{t} \tag{14}$$

Here, ℓ_0 is the initial size of the aggregate and t_0 the starting time of the growth of the aggregate. Based on this, we assume that $t_0 = 0$, which results in:

$$\frac{1}{2}\pi\left(\ell(t)^2 - {\ell_0}^2\right) = Dt \tag{15}$$

By rearranging the equation and solving for $\ell(t)$, we obtain:

$$\ell(t)^2 = \frac{2}{\pi}Dt + {\ell_0}^2 \tag{16}$$

$$\ell(t) = \sqrt{\frac{2}{\pi}Dt + {\ell_0}^2} \tag{17}$$

Equation (17) represents the general form of the empirical fit. Substituting the aggregate diameter d_a for ℓ yields the equation applied in MA:

$$d_a(t) = \sqrt{\frac{2}{\pi}Dt + {d_{a,0}}^2} \tag{18}$$

Supplementary Note 5: Particle Displacement

The displacements $\mathbf{r}_{j,n}$ of each individual particle n of the reference setup *Ref* are presented for the respective coordinates $j = (x, y, z)$ in Supplementary Figure 6. As indicated in Fig. 4 of MA, the expansion of the particles along the oscillation in the x-direction is twice as pronounced as in the y- and z-directions.

Supplementary Note 6: 1D Volume Fractions

The 1D volume fractions of all numerical variants are presented in Supplementary Figure 7 and complement the illustrations of the 2D volume fractions in the subfigures of Fig. 8 in MA. Each row of Supplementary Figure 7 represents one of the variants according to the respective tags, where the left panel presents the initial volume fraction and the right panel the outcome at the end of the simulations. The variants *Ref*, *HiAmp*, and *LoAmp* share the same initial arrangement. The representations of *Ref* are equivalent to the lower panel of Fig. 6 in MA and are shown here for the sake of completeness. No further explanations are provided here; please refer to the explanations in MA for Fig. 8.

Supplementary References

1. Jacobson, C. A. *International Space Station Remote Sensing Pointing Analysis* in *2007 IEEE Aerospace Conference* (IEEE, Big Sky, MT, USA, 2007), 1–13.
2. Bailey, A. E. *et al.* Spinodal Decomposition in a Model Colloid-Polymer Mixture in Microgravity. *Phys. Rev. Lett.* **99,** 205701 (20 2007).
3. Lu, P. J. *et al.* Orders-of-magnitude performance increases in GPU-accelerated correlation of images from the International Space Station. *JRTIP* **5,** 179–193 (2010).
4. Kleischmann, F., Luzzatto-Fegiz, P., Meiburg, E. & Vowinckel, B. Pairwise interaction of spherical particles aligned in high-frequency oscillatory flow. *J. Fluid Mech.* **984,** A57 (2024).
5. Uhlmann, M. An immersed boundary method with direct forcing for the simulation of particulate flows. *J. Comput. Phys.* **209,** 448–476 (2005).
6. Kempe, T. & Fröhlich, J. An improved immersed boundary method with direct forcing for the simulation of particle laden flows. *J. Comput. Phys.* **231,** 3663–3684 (2012).

7. Biegert, E., Vowinckel, B. & Meiburg, E. A collision model for grain-resolving simulations of flows over dense, mobile, polydisperse granular sediment beds. *J. Comp. Phys.* **340,** 105–127 (2017).

8. Vowinckel, B., Withers, J., Luzzatto-Fegiz, P. & Meiburg, E. Settling of cohesive sediment: particle-resolved simulations. *J. Fluid Mech.* **858,** 5–44 (2019).

9. Bergstöm, L. Hamaker constants of inorganic materials. *Adv. Colloid Interface Sci.* **70,** 125–169 (1997).

10. Israelachvili, J. N. Adhesion forces between surfaces in liquids and condensable vapours. *Surf. Sci. Rep.* **14,** 109–159 (1992).

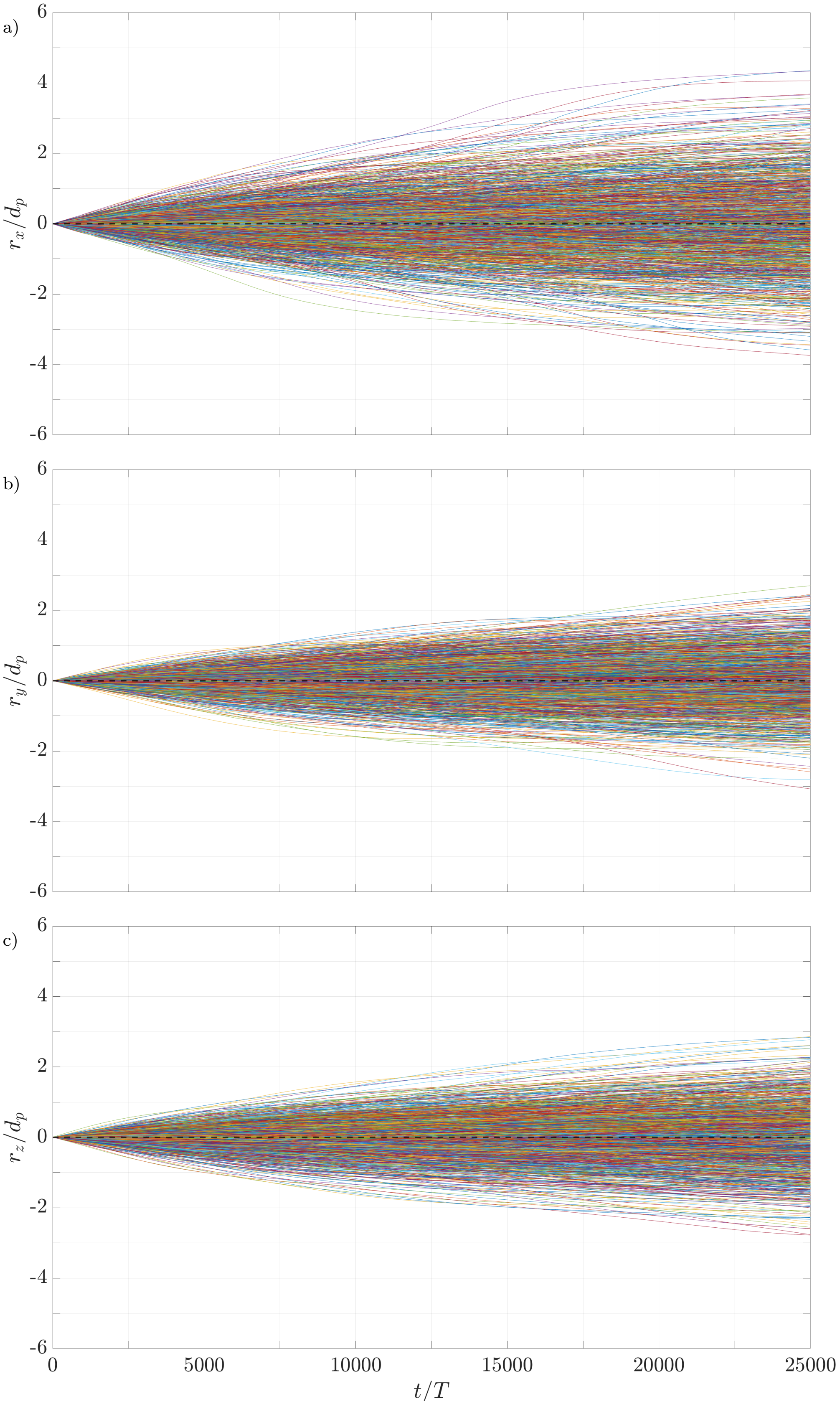

Supplementary Figure 6: Actual displacement $r_{j,n}(t)$ of each individual particle of the numerical reference setup *Ref.* a) presents the x-, b) the y-, and c) the z-coordinate direction.

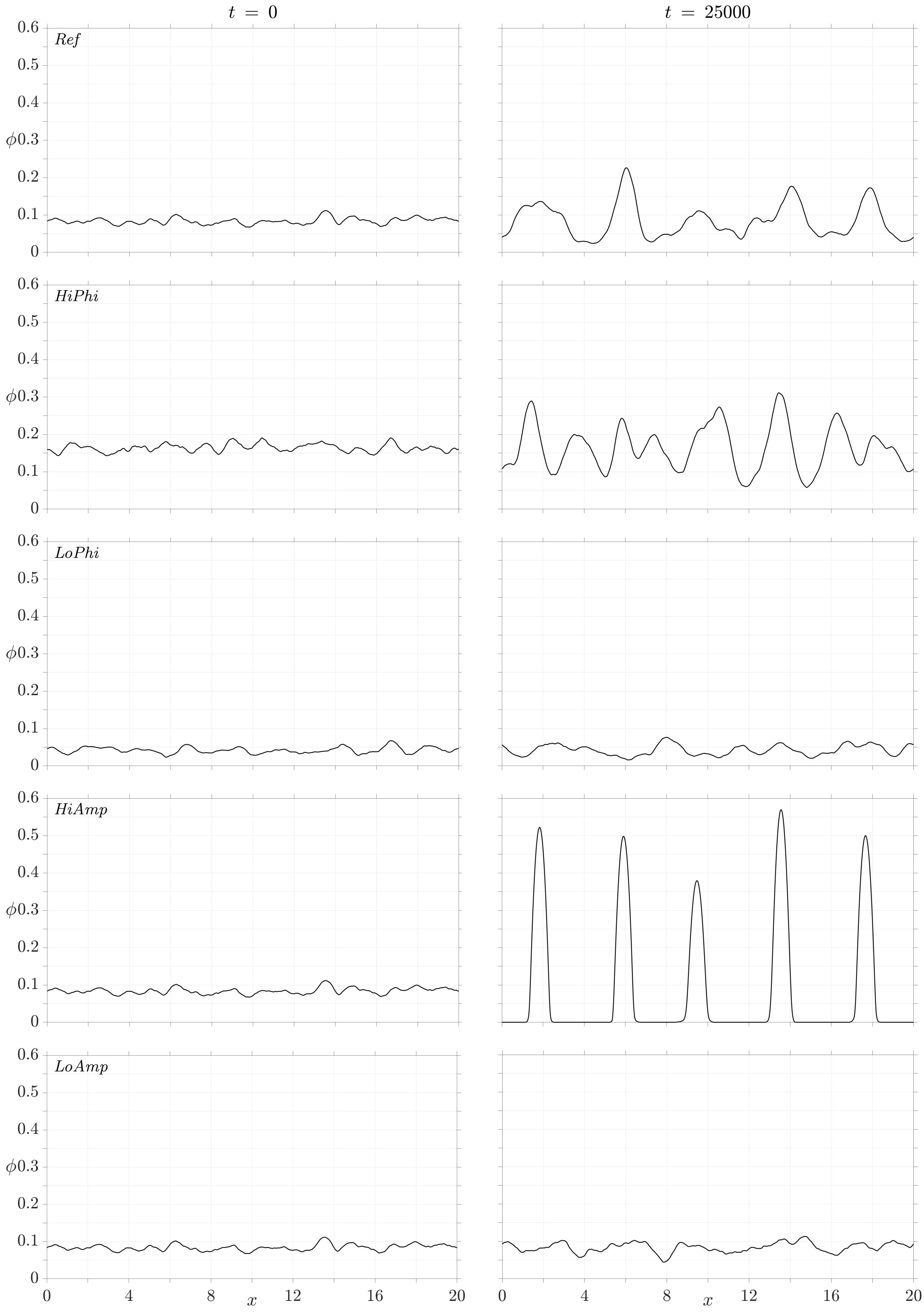

Supplementary Figure 7: 1D volume fractions of the numerical variants for the initial arrangement at $t = 0$ (left panel) and the final arrangement at $t = 25000$ (right panel).
